# Physiological and transcriptomic responses to cold waves of the most cold-tolerant mangrove, *Kandelia obovata*

**DOI:** 10.3389/fpls.2023.1069055

**Published:** 2023-02-10

**Authors:** Shanshan He, Xianxian Wang, Zhiyu Du, Pingping Liang, Yifan Zhong, Lin Wang, Yuan-Ye Zhang, Yingjia Shen

**Affiliations:** Key Laboratory of the Ministry of Education for Coastal and Wetland Ecosystems, College of the Environment and Ecology, Xiamen University, Xiamen, Fujian, China

**Keywords:** cold acclimation, mangrove, *Kandelia obovata*, cell wall modification, ubiquitination, CBF

## Abstract

Mangrove forests inhabit tropical or subtropical intertidal zones and have remarkable abilities in coastline protection. *Kandelia obovata* is considered the most cold-tolerant mangrove species and has been widely transplanted to the north subtropical zone of China for ecological restoration. However, the physiological and molecular mechanisms of *K. obovata* under colder climate was still unclear. Here, we manipulated the typical climate of cold waves in the north subtropical zone with cycles of cold/recovery and analyzed the physiological and transcriptomic responses of seedlings. We found that both physiological traits and gene expression profiles differed between the first and later cold waves, indicating *K. obovata* seedlings were acclimated by the first cold experience and prepared for latter cold waves. 1,135 cold acclimation-related genes (CARGs) were revealed, related to calcium signaling, cell wall modification, and post-translational modifications of ubiquitination pathways. We identified the roles of CBFs and CBF-independent transcription factors (ZATs and CZF1s) in regulating the expression of CARGs, suggesting both CBF-dependent and CBF- independent pathways functioned in the cold acclimation of *K. obovata*. Finally, we proposed a molecular mechanism of *K. obovata* cold acclimation with several key CARGs and transcriptional factors involved. Our experiments reveal strategies of *K. obovata* coping with cold environments and provide prospects for mangrove rehabilitation and management.

## 1 Introduction

Plants native to temperate zones undergo a period of temperature fluctuation between warm and cold seasons (Wang et al., 2019). Cold acclimation is used to describe the phenomenon by which plants increase their cold tolerance to cope with low temperatures (Guy, 1990). Cold acclimation can initiate universal metabolism adjustments, cell and tissue structure remodeling, transcriptional and post-translational regulations of plants for them to survive winter (Guy, 1990; Chinnusamy et al., 2007). Cold acclimation can be roughly split into three steps: 1) perception, which is the short-term perturbation in the metabolic homeostasis; 2) signal transduction, which is the memory of cold signals and experiences; 3) physiological and gene regulatory response, which is the establishment of a new homeostasis through reprogramming gene expression and regulating physiological state, which helps organisms to survive through the multiple cold waves in winter (Ensminger et al., 2006; Kazemi-Shahandashti and Maali-Amiri, 2018).

The regulatory network that functions in cold acclimation comprises several key sensors and regulators. Ca^2+^ is a secondary messenger and Ca^2+^ influx channels are considered important cold sensors in plants (Pareek et al., 2017). Calcium signals are transduced and decoded by calcium sensors, including calmodulin (CAM), CaM-like (CMLs), calcineurin B-like proteins (CBLs), and Ca^2+^ dependent protein kinases (CPKs), and then transmitted to the downstream processes (Pareek et al., 2017). C-repeat/DREB binding factors (CBFs) are crucial transcription factors in cold acclimation by regulating cold-regulated (COR) genes, many of which are demonstrated to enhance the freezing tolerance of plants (Thomashow, 1998). CBFs can bind to the CRT/DRE cis-element, containing the conserved ‘CCGAC’ motif to regulate COR expression (Shi et al., 2018). This ‘CBFs-COR genes’ responsive pathway is known as the CBF-dependent pathway (Ding et al., 2019). Recent studies also report that some CBF-independent transcription factors like ZAT10, ZAT12, HSFC1, ZF, and CZF1 were parallelly expressed with CBFs in cold response of *Arabidopsis thaliana*, and these genes were considered involved in the CBF-independent pathway in cold acclimation (Park et al., 2015, 2018).

The cell wall is a dynamic system and participates in almost all plant-environment interactions (Engelsdorf et al., 2019). Previous studies indicated that cold acclimation can lead to changes in the composition and structure of the cell wall (Takahashi et al., 2019). Wall mechanical integrity is monitored by the sensors of cell wall integrity (CWI), which locate in the plasma membrane and transmit signals into the cytoplasm to initiate cell wall remodeling (Novaković et al., 2018). Cell wall stiffening/loosening status can also be fine-tuned in response to cold stresses (Novaković et al., 2018). Furthermore, the post-translational process of ubiquitin is also considered to play an important role in response to cold stress (Miricescu et al., 2018). Ubiquitin is prevalent and highly conserved among eukaryotes (Zheng and Shabek, 2017) and catalyzed via sequential actions of three enzyme groups: ubiquitin-activating enzyme (E1), ubiquitin-conjugating enzyme (E2), and ubiquitin ligase (E3). At the end of the ubiquitination cascade, E3 recognizes target proteins for ubiquitination, where the target proteins will be degraded or be modified in response to varying environmental stress, including cold stress (Hondo et al., 2007; Chen and Hellmann, 2013; Swatek and Komander, 2016).

Mangrove forests distribute in the intertidal areas of the tropical and subtropical zones (Giri et al., 2010). They have tremendous ecological and economic values including coastline protection, carbon sequestration, providing nurseries for aquatic species, etc. (Schmitt and Duke, 2016). The area of the mangrove ecosystem declined globally over the past several decades, and mangrove restoration projects through afforesting had been carried out in many countries (Saintilan et al., 2020). Temperature is one limiting factor for mangrove growth and distribution (Ellison, 2001; Quisthoudt et al., 2012; Su et al., 2019). Most mangrove species are sensitive to chilling (0-15°C), and only a few species, including *Kandelia obovata*, *Avicennia marina*, *Aegiceras corniculatum*, are tolerant to such chilling temperature range.

*K. obovata* naturally distribute in Eastern Asia, from the Gulf of Tonkin northeastward to southern Japan (Sheue et al., 2003), is considered to be the most cold-tolerant mangrove species (Chen et al., 2010). The northernmost natural distribution of *K. obovata* in China is Ningde City, Fujian province (Chen et al., 2010), located at the junction of south subtropical and north subtropical zones (Fang, 2001), with typical cold waves in autumn and winter. The lowest monthly average temperature in Ningde (26°18′-27°40′N, 118°32′-120°43′E) is 10.3°C; and the lowest temperature can be as low as 4.2°C ^1^. Owing to its cold tolerance, *K. obovata* is often employed for mangrove restoration, in particular in the north subtropical zones (Liu et al., 2018).

The low temperature commonly leads to metabolic disorders of *K. obovata*, such as leaf scorch and massive leaf fall (Wang et al., 2011), limited photosynthesis (Liu et al., 2018), increased reactive oxygen species (ROS) content (Peng et al., 2015), etc. However, previous experience in transplantation suggested that exposing *K. obovata* seedlings to chilling temperature before transplanted significantly enhances the survival of seedlings (Liu et al., 2018), indicating cold experience can improve the cold tolerance of *K. obovata*. These studies involved only one cold wave, and the molecular mechanisms of cold acclimation involving repeated cold waves remain unknown.

**Figure 1:**
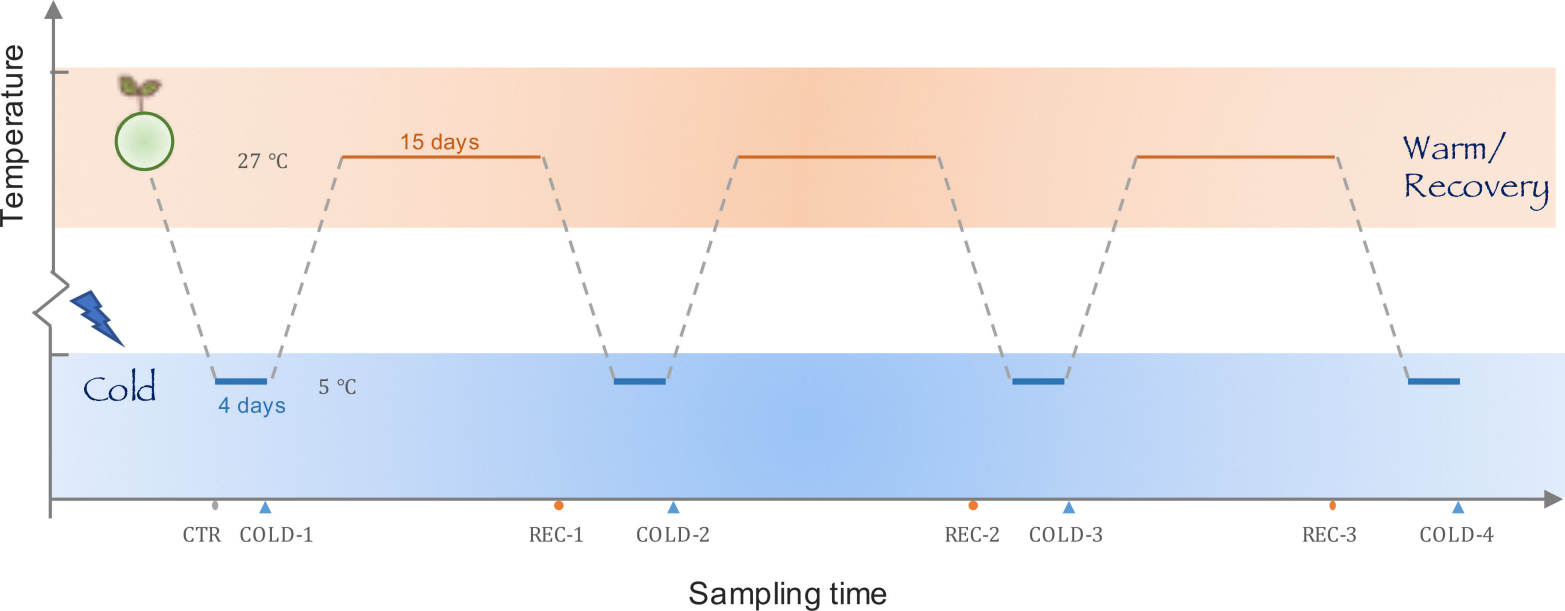
The design of the cold wave experiment. We mimicked the typical winter climate on the coast of the north subtropical zones with three cold-recovery cycles, each cycle consisted of a 4-day cold treatment at 5°C and a 15-day recovery treatment at 27°C. Samples for physiological and transcriptome analysis were collected before the cold treatment, and at the end of each cold and recovery treatment.

To explore the physiological and molecular mechanisms of subtropical plants to colder climates, we mimicked the typical climate of cold waves in the north subtropical zone and treated *K. obovata* seedlings with three cycles of cold stress and recovery (**Figure 1**). In the experiment, we measured physiological and transcriptomic responses of seedlings under each cold stress and recovery stage. Different response patterns between first cold wave and repeat cold waves were observed for both physiological indexes and transcriptional expression profile, in accord with the process of cold acclimation. Therefore, we hypothesized that a typical cold acclimation mechanism was adopted by *K. obovata* seedlings in response to repeat cold waves. Our study contributes novel findings regarding the environmental adaptation in subtropical plants and warrants future researches on practices of improving mangroves’ cold tolerance in plantations.

## 2 Materials and methods

### 2.1 Sampling and seedlings preparing

Propagules of *K. obovata* were picked from Zhangjiangkou Mangrove National Nature Reserve in March 2017 (Fujian, China; 23°53'-23°56'N, 117°24'-117°30'E), the natural habitats of *K. obovata* (Sheue et al., 2003; Su et al., 2019). Propagules were potted in the sand, and the base of propagules was immersed with Hogland’s solution and 10‰ artificial seawater. Propagules grew into seedlings in a plant culture room, with 27/22°C (day/night) and 16/8 h (day/night) photoperiod, the photosynthetic photon flux density (PPFD) was 130μmol m^-2^ s^-1^. All plants used in our experiments were cultivated under the same conditions. The *K. obovata* seedlings were cultivated from March 2017 to February 2018 and were then used in the experiment.

### 2.2 Pre-experiment

The exact cold acclimation temperatures are species-dependent (Kazemi-Shahandashti and Maali-Amiri, 2018). An appropriate cold acclimation temperature should ensure the survival of plants during the experiment exposure. To find the appropriate temperature for cold treatment, we performed a test experiment, where three groups of seedlings were put into 0 ± 1°C, 5 ± 1°C and 10 ± 1°C low-temperature incubators for 24 hours. In this test experiment, we found that seedings died within a week under 0 ± 1°C, but survived under 5 ± 1°C and 10 ± 1°C. We therefore adopted 5°C in the cold acclimation experiment.

### 2.3 Cold waves

The cold wave was manipulated as a four-day cold treatment with 5 °C followed by a 15-day recovery treatment at 27°C (**Figure 1**). The experiment included four cold treatment (COLD-1/2/3/4) and three recovery treatments (REC-1/2/3).

In the experiment, six propagules of *K. obovata* were planted in three pots (individuals 1 and 4 in pot I, individuals 2 and 5 in pot II, and individuals 3 and 6 in pot III) and grew into seedlings in a warm plant culture room (27°C). Besides samples for each treatment (COLD-1/2/3/4, REC-1/2/3), we collected samples from individuals 1-6 before any treatment was given as controls (CTR). At the end of each treatment, leaf samples for physiological analysis were collected from individuals 1-3 and leaf samples for transcriptomic analysis were collected from individuals 4-6. Samples for both analyses were collected from the same pots to reduce the variation in the physiological and transcriptomic analyses. The experiment was conducted in two growth rooms. Each time during cold treatment (COLD-1/2/3/4), the plants were moved to the cold culture room (5°C) for 4 days. During recovery phases (REC-1/2/3) and CTR, the seedlings grew in the warm culture room (27°C). In addition to the difference in temperature between our cold culture room and warm culture room, other culture conditions were identical. The photoperiod in both cold and warm treatment periods was changed to 14/10 h (day/night) to simulate the shortened duration of illumination in autumn and winter.

### 2.4 Physiological tests

Plants exposure to cold stress will impair the cellular metabolism and lead to accumulated reactive oxygen species (ROS), including superoxide radical anions (O^2-^) and hydrogen peroxide (H_2_O_2_) (Novaković et al., 2018). Malondialdehyde (MDA) is the final product of lipid peroxidation, which can be used as a measurement of oxidative damage under cold stress, research in *Magnolia denudata* found that the MDA concentration increased with the enhancement of the cold acclimation (Wu et al., 2021). Antioxidant enzymes, including superoxide dismutase (SOD) and peroxidase (POD), can protect proteins, metabolites, and cell structures against oxidation and peroxidation (Hincha and Zuther, 2020). SODs catalyze oxidation and reduction of superoxide anions and generate H_2_O_2_, a stable type of ROS, and PODs are efficient in reducing H_2_O_2_ to H_2_O. In this experiment, content of MDA, activities of SOD and POD, and content of chlorophyll of leaves were measured to evaluate the physiological states of *K. obovata* seedlings.

Leaves of *K. obovata* seedling individuals 1, 2, and 3 were stored in the icebox and tested immediately by using the reagent kits according to the manufacturer’s instructions (Jiancheng Co., Ltd., Nanjing, A003-1, A001-1, A084-3, A147-1).

The difference of physiological indicators between phases was compared using one-way ANOVA with *p* values of < 0.05 considered to be significant.

### 2.5 RNA-seq data analyses

Leaves of *K. obovata* seedling individuals 4, 5, and 6 were rapidly frozen in liquid nitrogen and then moved to the -80°C refrigerator and then sent to the Experimental Department of Novogene Co. (Beijing, China) for total RNA extraction, library construction, and RNA-sequencing. Initial quality control was done using FastQC (V 0.11.9) followed by trimming with Trimmomatic (V 0.38) (Bolger et al., 2014) to remove adapters and low-quality base pairs. The selection of trimming steps and their associated parameters are supplied on the command line (ILLUMINACLIP:/Trimmomatic-0.38/adapters/TruSeq3-PE-2. fa: 2: 30: 10: 1: true HEADCROP: 12 LEADING: 3 TRAILING: 3 SLIDINGWINDOW: 4: 20 MINLEN: 50). These clean reads were subjected to downstream analyses.

Paired-end clean reads were aligned to the reference *K. obovata* genome (Qiao et al., 2020) using HISAT2 (V 2.1.0) (Kim et al., 2019). FeatureCounts (Liao et al., 2013) was used to count the reads numbers mapped to each gene. Before differentially expressed gene analysis, genes with a read count smaller than ten in all samples combined were removed from further analyses. The R package “DESeq 2” (Anders and Huber, 2010) was used to analyze the differentially expressed genes (DEGs). Genes with at least |log2FoldChange| > 2 and padj < 0.01 were designated as differentially expressed. All genes with expression at any phase in the experiment were used for Principal Component Analysis (PCA). PCA was performed using the packages DESeq2 and ggplot2 after variance stabilizing transformation of the count data by the function of DESeq2.

### 2.6 Definition of cold acclimation-related genes

To know the cold acclimation mechanism of *K. obovata* seedlings, we defined cold acclimation-related genes (CARGs) based on the analysis results of DEGs. We set CTR, REC-1, and REC-2 as ‘Warm’ status, genes differentially expressed (|log2FoldChange| > 2 and padj < 0.01) between COLD-1/Warm versus COLD-2/Warm or COLD-4/Warm were named CARGs.

### 2.7 GO enrichment analysis

Gene ontology enrichment analysis (Ashburner et al., 2000) was performed using the OmicShare tools, a free online platform for data analysis^2^, FDR was calculated using the hypergeometric test. All CARGs were submitted to do the GO enrichment analysis, with FDR <0.05 as the threshold, GO terms satisfying this condition were defined as GO terms that were significantly enriched.

### 2.8 Identification of potential koCBFs target genes

To investigate the possible *koCBF*s target genes (Shi et al., 2018), a Perl script (Supplementary Information 1) was used to extract the upstream 2000 bp putative gene promoter regions before the start codon of all *K. obovata* genes to find the existence of the CRT/DRE motif.

### 2.9 Co-expression network analysis of DEGs and physiological indexes

WGCNA R package (Version 1.69) (Zhao et al., 2010; Liang et al., 2020) was adopted for the co-expression network analysis. All 2,681 DEGs were used to construct a signed co-expression network. The soft-thresholding power value was chosen as empiric value as 18 dues to no suitable estimated soft power value.

The biological meanings of the eigengenes can be further analyzed by calculating the Pearson correlation of physiological indices data with the eigengene of each module. We used the absolute value of the Pearson’s correlation of Module Membership (MM) and Gene Significance (GS) to select hub genes (Zhao et al., 2010; Liang et al., 2020). Considering the potential cold acclimation-related biological functions of genes in Modules turquoise, blue and yellow, we identified hub genes within-module by combining GS of POD and SOD and MM of Modules turquoise, blue and yellow. Hub gene sets in different modules were identified with the criteria: | MM | > 0.8 and | GS | > 0.3. Hub-transcription factors were manually screened based on gene annotations. In addition, it has been reported that there are a series of genes in Arabidopsis that are co-regulated with *ATCBF*s and co-regulate the cold acclimation process, they are called “First Wave” genes (Park et al., 2018). Based on this report, we also found transcription factors that were expressed in parallel with *koCBF4* in our data.

## 3 Results

### 3.1 Physiological and transcriptional responses to cold waves

**Figure 2:**
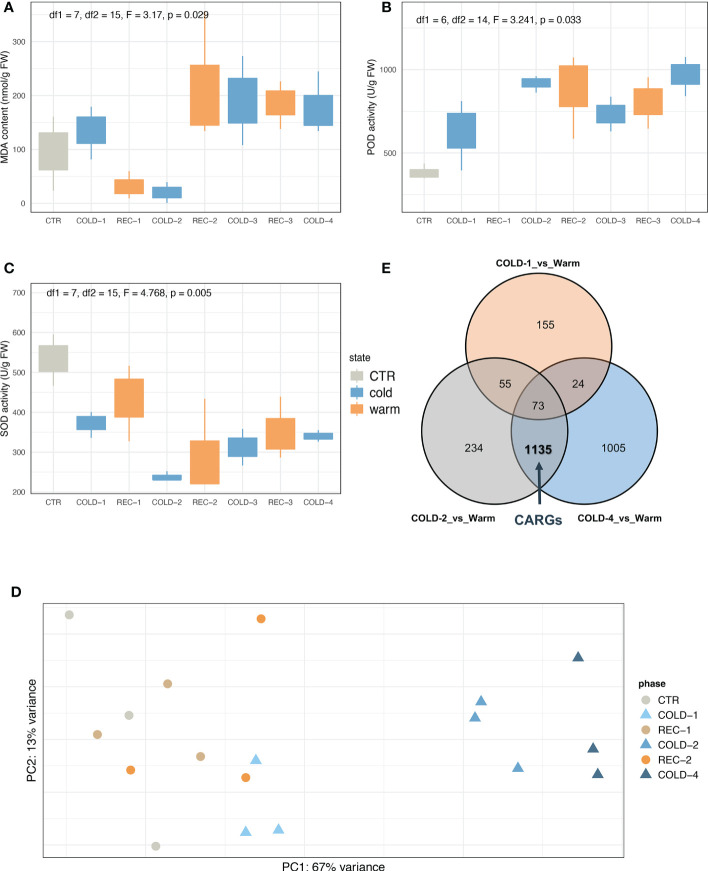
Physiological and transcriptomic responses of *K. obovata* seedlings to circles of cold and recovery. Enzyme activity changes of malondialdehyde (MDA) **(A)**, peroxidase (POD) **(B)**, and superoxide dismutase (SOD) **(C)**. **(D)** PCA results of transcriptome analysis. Circles mean warm conditions (CTR and REC-1/2), triangles mean cold treatments (COLD-1/2/4). **(E)** The Veen plot of 2,681 DEGs between the cold treatments (COLD-1/2/4) and the warm conditions (CTR and REC-1/2). The bold value represented the number of cold acclimation related genes (CARGs). The definition of CARGs was based on the analysis results of DEGs. CTR, REC-1, and REC-2 were set as ‘Warm’ status, genes differentially expressed (|log2FoldChange| > 2 and padj < 0.01) between COLD-1/Warm versus COLD-2/Warm or COLD-4/Warm were named CARGs.

We found that cold waves significantly changed seedlings’ malondialdehyde (MDA) content (F = 3.17, *p* = 0.029), superoxide dismutase (SOD) enzyme activity (F = 4.768, *p* = 0.005), and peroxidase (POD) enzyme activity (F = 3.241, *p* = 0.033) (**Figures 2A-C**, see **Supplementary Figure 1** for the scatterplots showing the specifics of each sample). The SOD enzyme activity decreased in the first two cold waves but increased slightly in the latter waves. The POD enzyme activity showed a minor increase in the first cold wave but a greater increase in the later cold waves. Compared to the control, the MDA content only decreased in the second cold wave and appeared to recover in subsequent cold waves. Chlorophyll contents also tended to differ between the first two and the subsequent cold waves (**Supplementary Figure 2**).

The transcriptome sequencing generated an average of 26 million paired-end reads from each of the 18 samples (**Supplementary Table 1**). These samples were clustered by the principal component analysis (PCA) into two groups (**Figure 2D**). One group includes samples from the first cold treatment (COLD-1) and warm conditions (CTR and recovery phases), and the other included samples from the later cold waves (COLD-2 and COLD-4). Consistent with the PCA results, the *k*-means analysis also showed that COLD-1 can be separated from COLD-2 and COLD-4 (*k*=2, **Supplementary Figure 3**). Also, the differentially expressed genes (DEGs) between COLD-1 and CTR were less than the other two comparisons (COLD-2 vs. REC-1 and COLD-4 vs. REC-2, **Supplementary Figure 4**). These results suggested that seedling responses differed between the first and later cold waves.

The first cold experience initiated the cold acclimation process, and the later cold waves manipulated the scenario to show the cold acclimation effects. Both PCA and *k*-means (**Figure 2D**; **Supplementary Figure 3**) results showed that CTR, REC-1 and REC-2 samples were clustered with their replicates overlapping. Thus we merged CTR, REC-1 and REC-2 as ‘Warm’, and defined genes whose expression was not affected by the first cold (not DEGs in COLD-1 vs. Warm) but significantly affected by the later cold (DEGs in both COLD-2 vs. Warm and COLD-4 vs. Warm) as cold acclimation-related genes (CARGs) (**Figure 2E**, see details in **Method** ). This defined analysis revealed 1,135 CARGs (**Supplementary Table 2**), and 3/4 CARGs were upregulated in COLD-2 and COLD-4 treatments, referred to as upregulated CARGs (**Supplementary Figure 5**). GO enrichment analyses showed that CARGs were significantly enriched (FDR < 0.05, **Supplementary Figure 6**) in pathways related to transcription factor activity, oxidation-reduction process, calcium ion binding, protein ubiquitination, and cell wall.

### 3.2 Calcium signaling, cell wall modification, and ubiquitination processes in the cold acclimation

**Figure 3:**
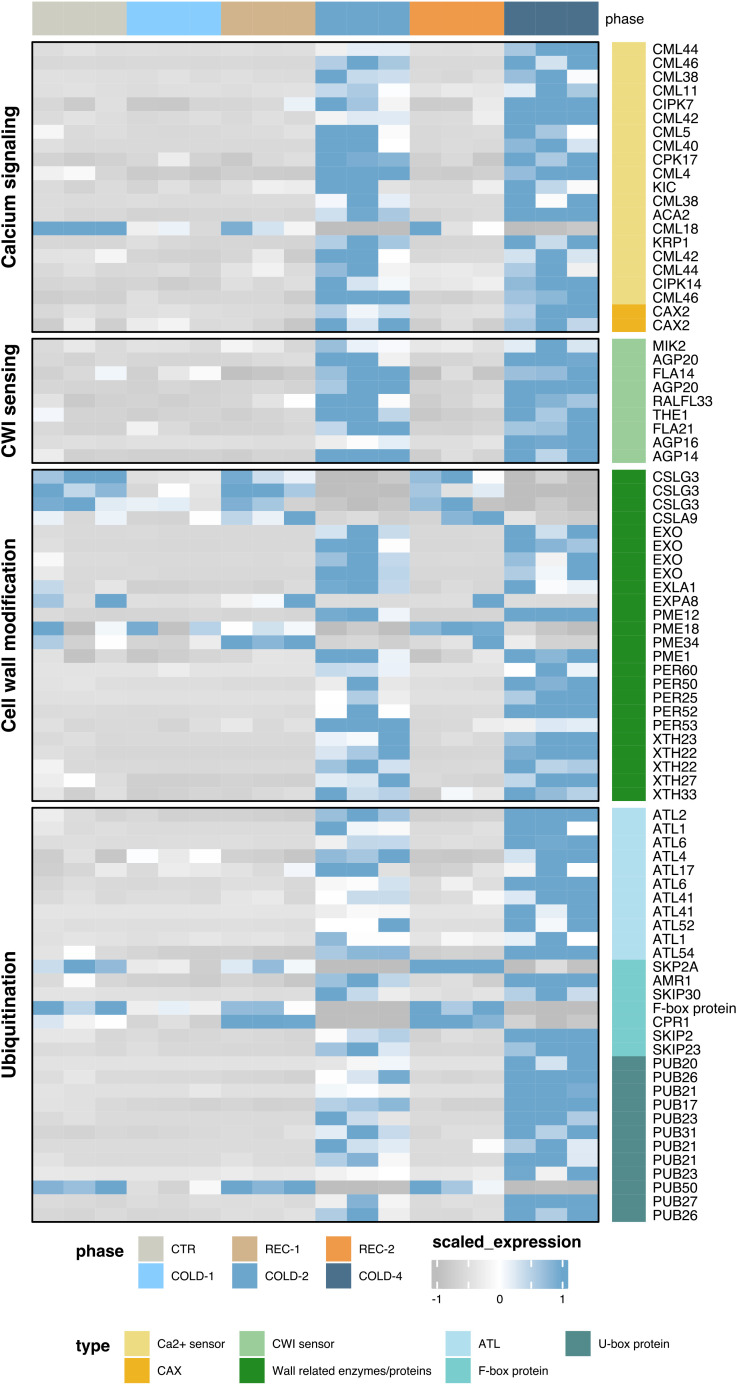
The expression heatmaps of CARGs involved in the calcium signaling, cell wall modification, and ubiquitination pathways.

In the calcium ion binding pathway, we found that two Vacuolar cation/proton exchanger 2 (*CAX2*) genes were upregulated CARGs (**Figure 3**), which are Ca^2+^/H^+^ exchangers (Kazemi-Shahandashti and Maali-Amiri, 2018). Genes encoding calcium sensors, including *CPK*s, *CIPK*s, and *CML*s, were also upregulated CARGs (**Figure 3**), but *CML18* was downregulated CARGs. In the cell wall pathway, we found that previously reported cell wall integrity sensors are CARGs in our list, including the arabinogalactan-proteins (*AGP*s), Fasciclin-like AGPs (*FLA*s), one Rapid alkalinization factor (*RALFL33*), and two LRR receptor-like serine/threonine-protein kinases (LRR-RLKs), *MIK2*, and *THE1* (**Figure 3**) (Johnson et al., 2003; Cheung and Wu, 2011; Novaković et al., 2018). In particular, *RALFL33* belonged to both in calcium-dependent signaling network and cell wall integrity, suggesting cold acclimation of *K. obovata* involves signaling between calcium and cell wall integrity sensors.

Consistent with the physiological responses of peroxidase (**Figure 2B**), we found that five class III peroxidase superfamily protein genes (*PER*s) were upregulated CARGs (**Figure 3**). CARGs also included four cellulose synthases like (*CSL*s) genes, five xyloglucan endotransglucosylase/hydrolase (*XTH*s) genes, four pectin methylesterase (*PME*s) genes, four EXORDIUM (*EXO*s) genes, and two Expansin-like genes, *EXLA1* and *EXPA8*. The products encoded by these genes (Novaković et al., 2018) are regulators in cell wall modification affecting the hardness or softness of cell wall.

Most CARGs enriched in the protein ubiquitination process encoded E3 ligases (**Figure 3**), including 11 Arabidopsis thaliana Toxicos en Levadura (*ATL*s), seven F-box protein genes, and 12 Plant U-box type E3 ubiquitin ligases (*PUB*s). Among these E3 ligases, ATL54 was previously reported to function in secondary cell wall formation (Noda et al., 2013). These results thus suggest a connection between ubiquitination regulation and cell wall modification underlying cold acclimation mechanisms of *K. obovata*.

### 3.3 Co-expression analysis and key transcription factors identification

**Figure 4:**
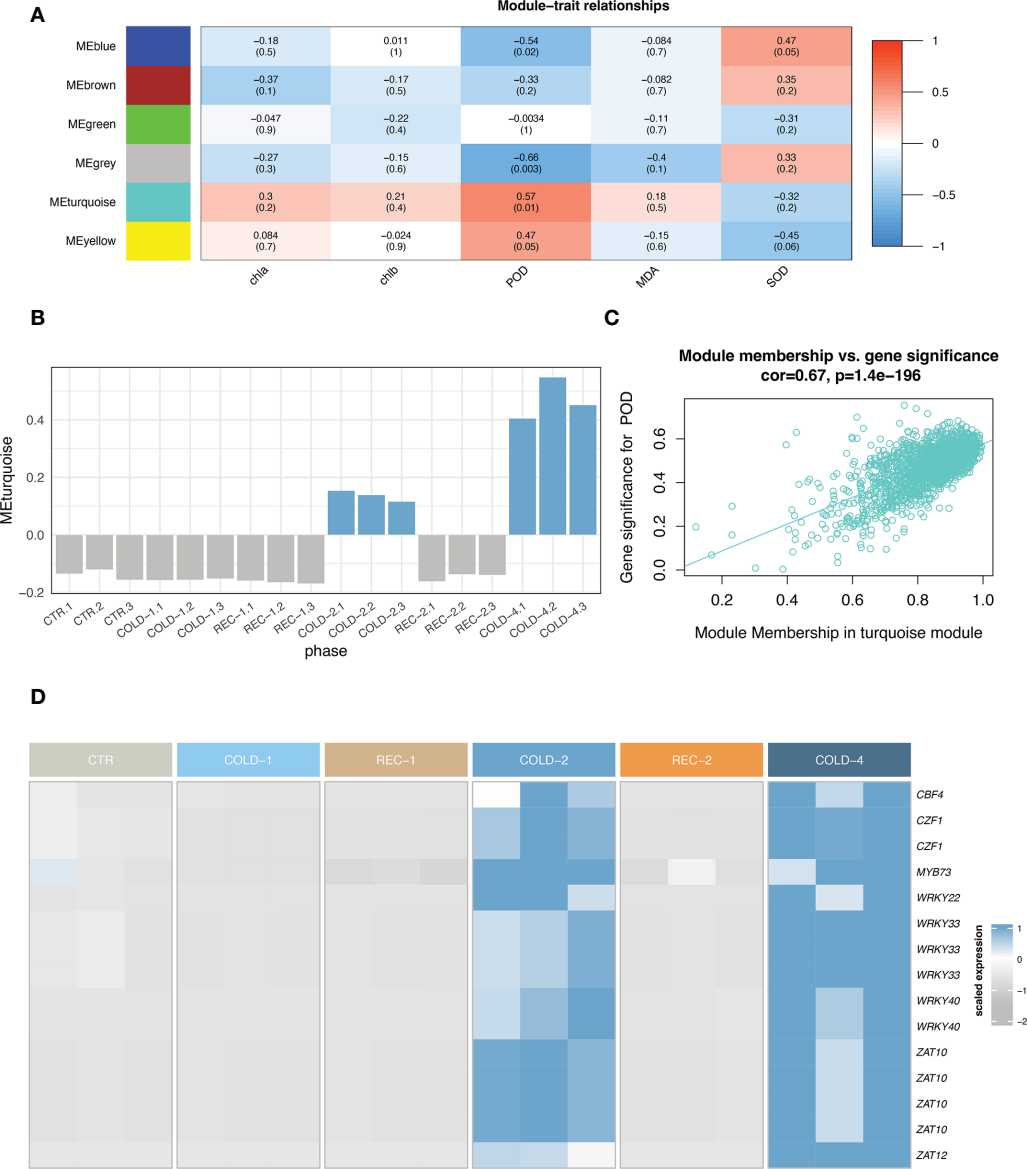
Co-expression network of DEGs. **(A)** Heatmap of the module-trait relationships. The numbers in a cell were the Pearson correlation coefficient between the module eigengene and the trait, and the numbers in the bracket showed the p values. The figure was color-coded by correlation coefficient. MDA, malondialdehyde; SOD, superoxide dismutase; POD, peroxidase; chla, chlorophyll a content; chlb, chlorophyll b content. **(B)** The eigengene expression profile in Module turquoise. **(C)** The gene significance of POD against turquoise module membership. **(D)** The heatmap of representative hub-transcription factors.

Co-expression network analysis with all 2,681 DEGs (**Figure 2E**) and the physiological indices data revealed six modules (**Figures 4A**; **Supplementary Figure 7**), including Module turquoise (1,504 genes), blue (563 genes), brown (306 genes), yellow (101 genes), green (77 genes), and grey (130 genes). The eigengene of a module represents a consensus expression pattern of genes in this module (Zhao et al., 2010; Liang et al., 2020). The Eigengenes of Modules turquoise, blue and yellow are similar to the expression pattern of CARGs (**Figure 4B**; **Supplementary Figure 8**). Eigengenes in Module turquoise and yellow were positively correlated with POD, eigengene in Module blue was positively correlated with SOD (**Figure 4C**; **Supplementary Figure 9**), which means that the gene expression patterns and physiological parameters covaried for the three modules.

From these three modules (turquoise, blue and yellow), we identified 1,387 hub genes (**Figure 4**; **Supplementary Figure 10**). 777 (56%) hub genes were also CARGs (**Supplementary Figure 10B**, **Supplementary Table 2**), and 75 were hub-transcriptional factors (**Supplementary Table 2**). These hub-transcriptional factors included C-repeat/DREB binding factors (CBFs) and were classified into seven families: bHLHs (4), DREBs (3), NACs (5), ERFs (16), MYBs (10), WRKYs (16), and ZATs (8).

CBFs were previously demonstrated to be crucial transcription factors that enhanced the freezing tolerance of plants (Thomashow, 1998). We identified four copies of *CBF*s in the *K. obovata* genome, among which *koCBF4* was a hub-transcription factor, *koCBF3* was CARGs and the other two *koCBFs* were also upregulated in the repeated cold treatments (**Supplementary Figure 11**, **Supplementary Table 2**). To identify the genes potentially regulated by *koCBFs*, we screened the 1,135 CARGs and found 491 (43%) genes have at least one CBF binding motif (**Supplementary Table 3**, **Supplementary Figure 12**). Thus, *koCBFs* may play a critical role in regulating the cold acclimation co-expression network.

We also found 14 hub-transcription factors expressed in parallel with *koCBF4* (**Figure 4D**, see details in **Method**), including *MYB73*, *WRKY22*, *ZAT12*, two *CZF1*s, two *WRKY40*s, three *WRKY33*s, and four *ZAT10*s. CZF1, ZAT10, and ZAT12, they were previously recognized in *Arabidopsis thaliana* as the CBF-independent transcription factor in cold responses (Park et al., 2018). The parallel expression of these hub-transcription factors and *koCBF4* suggests that the transcriptional regulation underlying *K. obovata* cold acclimation involved both the CBF-dependent and CBF-independent pathways.

## 4 Discussion

In this study, we treated mangrove plants with the cold waves mimicking the winter of the northern subtropical region and found that the physiological and transcriptional response of *K. obovata* seedling is generally different between the first and the later cold waves. The underlying gene regulation involved calcium signaling, protein ubiquitination, cell wall modification process, and several key transcriptional factors. In the following discussion, we will connect the key genes involved in these functions and develop a potential molecular mechanism underlying the adaptive response to cold waves of *K. obovata*.

### 4.1 The cold acclimation of *K. obovata* seedlings

**Figure 5:**
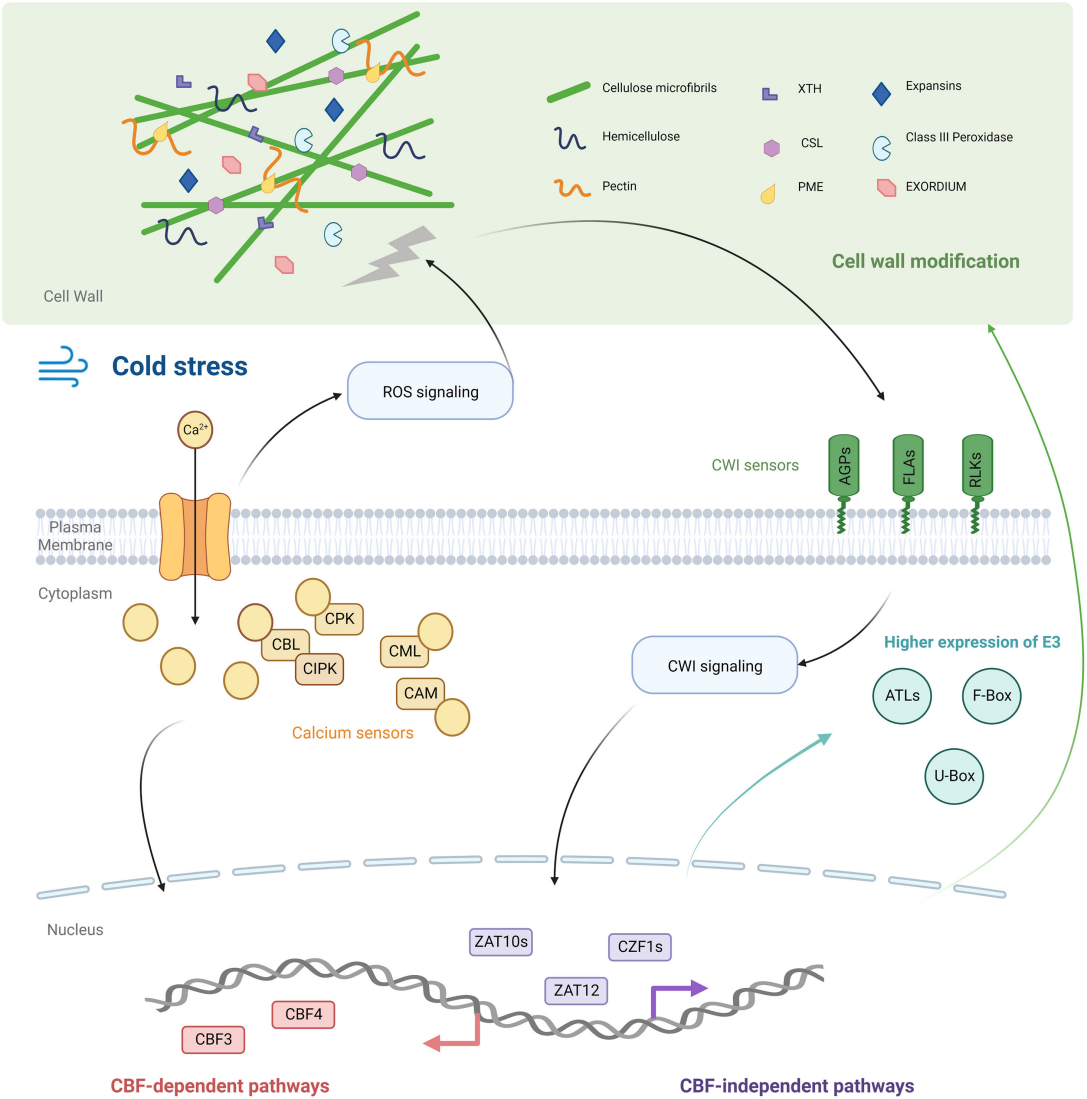
Schematic diagram of the cold acclimation mechanism of *K. obovata*. Cold stress elevated the cytosolic Ca^2+^ concentration to generate Ca^2+^ signals that were decoded by various calcium sensors. The influx of Ca^2+^ led to the production of ROS, which destroyed the cell wall integrity (CWI). The CWI sensors (AGPs and FLAs) perceived this signal and initiated cell well modification. Genes encoded proteins like PERs, CSLs, XTHs, PMEs, and EXOs modulated wall stiffening/loosening status. *K. obovata*’s Ub system may participate in cold acclimation via recruiting many E3 ligases including ATLs, F-box type, and U-box type E3 ligases. Ca^2+^ was upstream of the transcription factor CBFs, and *ko*CBFs regulated the expression of Cold Acclimation Related Genes (CARGs). Transcription factors in CBF-independent pathways (CZF1 and ZATs) were also hub transcription factors. Thus, CBF-dependent and -independent pathways co-regulated the cold acclimation of *K. obovata* seedlings.

We found that both the physiological and transcriptional responses of *K. obovata* seedlings differed between the first and subsequent cold waves. The MDA content dropped in the second cold wave but increased to a higher level in the third and fourth cold treatments. Another article exploring the mechanism of short-term cold acclimation of *K. obovata* found that, when seedlings were subjected to cold stimulus for the second time, the MDA content of seedlings have undergone a single cold treatment (no recovery) is significantly lower than that of seedlings without cold memory (Liu et al., 2018). Previous knowledge (Novaković et al., 2018) shows that the influx of Ca^2+^ can produce Reactive Oxygen Species (ROS), and the accumulation of superoxide can oxidize polyunsaturated fatty acids in the cell plasma membranes to produce MDA. Thus, MDA content is an important indicator of membrane lipid peroxidation, and SOD and POD are important enzymes in detoxifying ROS. In this study, the changing of MDA content and SOD enzyme activity showed different expression trends in the first half and the second half of our long-term cold waves experiment, the levels of MDA and SOD tend to increase after an initial decrease. It was worth mentioning that the changes in the expression of *PER*s (class III peroxidase superfamily protein genes) were very consistent with the changes in POD enzyme activity that we detected. *PER*s were significantly up-regulated (**Figure 3**), and the activity of the POD enzyme was also increased (**Figure 2D**). This consistency showed that the POD enzyme does play a role in the cold acclimation of *K. obovata*. However, despite the elevated tendency of SOD and POD enzyme activities, a high level of MDA indicated that the ROS effect had not been completely removed. MDA content presents a stable level in COLD-3 and COLD-4 might be explained by the retention of ROS, which can be used as a necessary signal transduction mechanism to promote cold acclimation (**Figure 5**).

Temperature fluctuations usually affect the photosynthetic efficiency of plants (Yuan et al., 2021). In our experiment, although the chlorophyll contents did not show strong changes (**Supplementary Figure 2**), we found that several genes related to photosystem were significantly downregulated, especially in COLD-4 (**Supplementary Figure 13A**). The proteins encoded by these genes are important components of each subunit in the photosynthetic system, among them, PSAs and PSBs are important subunits of photosystem II complex (PSII) and photosystem I complex (PSI), respectivily. Through the schematic diagram of plant photosystem (**Supplementary Figure 13C**), we found that almost one third of subunits of PSI and PSII were affected (**Supplementary Figure 13B**). This result showed that even though the temperature of each cold treatment was the same, the response of *K. obovata* seedlings to the regular cold waves was different in the early and late stages. The differential expression of genes related to the photosystem was also found in the study of *Brassica campestris*’s response to temperature fluctuations (Yuan et al., 2021). A previous study (Liu et al., 2018) also found that cold stress can disturb leaf photosynthesis of *K. obovata* seedlings. Reducing the photosynthesis rate may help *K. obovata* seedlings survive better, reflecting the necessity of cold acclimation for mangrove plants to survive the winter. However, this conclusion warrant further investigation.

In the gene expression part, the expressional profile did not show significant changes under the first cold wave; but about ten times more genes were differentially expressed in the later cold stress. Different responses between first and latter cold waves suggested that the first cold experience may alter the gene expression in later cold stress. This phenomenon is consistent with the concept of cold acclimation that the first cold experience can reprogram the response to subsequent cold events (Preston and Sandve, 2013), which is considered an adaption to cold climate.

### 4.2 Signal transduction in the cold acclimation of *K. obovata* seedlings

We found that cold acclimation genes (CARGs) enriched in the calcium signaling pathway included two cation/proton exchangers (*CAX2*) and 21 Calcium sensors (i.e. *CPK*s, *CIPK*s, and *CML*s). This observation is consistent with previous studies showing that cytosolic Ca^2+^ concentration was elevated under emergent cold stress and captured by calcium sensors (Yuenyong et al., 2018), which were upstream to CBFs in cold response signaling (Hiraki et al., 2018). As CARGs represented genes whose expression remained unchanged in the first cold wave but changed significantly during later waves, these results indicated that the Ca^2+^-mediated signaling pathway was not only involved in the first cold exposure but also in generating strong signals in later cold waves (**Figure 5**).

Besides the calcium signaling pathway, we found CARGs also enriched in the cell wall modification pathways. These genes include nine cell wall integration (CWI) sensors (e.g. *AGP*s, *FLA*s, *THE1*, *RALFL33*, and MIK2) and genes regulating the cell wall components (*CSL*s, *XTH*s, *PME*s, *EXO*s, Expansins, and *PER*s). The primary cell walls of plants are composed of three main components: cellulose, hemicellulose, and pectin. Cellulose microfibrils are embedded in a matrix of hemicelluloses and pectin, and changing the relative composition and connection between them gives the cell wall flexibility (Novaković et al., 2018). Cell wall status is monitored by CWI sensors, which are located in the plasma membrane. In this study, the enrichment of CWI sensors is consistent with the previous studies that cold treatment destroyed the mechanical properties of the cell wall, which was captured by CWI sensors (Johnson et al., 2003; Seifert and Roberts, 2007). THE1 and RALFL33 are known to detect the fragments of the cell wall, MIK2 senses the cellulose integrity, and AGPs and FLAs involve in stress-related wall remodeling (Novaković et al., 2018). In addition, a previous study suggested that ROS could cause cell wall damage (Novaković et al., 2018) and trigger the cell wall remodeling process initiated by CWI sensors, but details of this pathway remained to be explored.

After CWI sensors perceived the cell wall damage, the cell wall modification process may be initiated (Novaković et al., 2018). Our findings were consistent with previous studies that show alterations in these enzymes’ activities enhanced cold tolerance by affecting wall properties (Novaković et al., 2018). All these genes (*CSL*s, *XTH*s, *PME*s, *EXO*s, Expansins, and *PER*s) were demonstrated to be involved in cell wall flexibility (stiffening/loosening). CSLs and XTHs are found to influence the cell wall ingredient of hemicellulose, PMEs can regulate pectin stiffness/viscosity, EXOs and Expansins can control cell expansion, and PERs can affect the chemical bonds that connect components of the cell wall (Novaković et al., 2018; Cao et al., 2019; Takahashi et al., 2020). However, as these genes are involved in both cell wall stiffening and loosening processes (Novaković et al., 2018), whether the cell wall gets stiffer or looser in cold acclimation remains unknown (**Figure 5**).

Transcriptional binding sites analysis suggested that ten cell wall stiffening/loosening regulating genes (e.g. *PER*s and *XTH*s) had ‘CCGAC’ binding motif and maybe under the transcriptional regulation of *koCBF*s (**Supplementary Table 2**). This finding is consistent with previous studies on *Arabidopsis thaliana* and tea plant, *Camellia sinensis*, that cell wall modification genes were targets of CBFs during cold acclimation (Zhao et al., 2016; Wang et al., 2019). The results indicated that the cold acclimation of *K. obovata* seedlings may involve CBF-related signal transmission from calcium sensors to cell walls (**Figure 5**).

We found that CARGs were also enriched in pathways relating to Ubiquitination (**Supplementary Figure 6**). The genes enriched in these pathways included three kinds of E3 ligases, ATLs, F-boxes, and U-boxes. Previous knowledge shows that these E3 ligases are important in substrate identifying and binding, in mediating cell division, nutrition balance, and plant growth (Song et al., 2017; Miricescu et al., 2018). These studies have emphasized the key role of ubiquitination in regulating plant development and acclimating to environmental changes, through mediating the proteolysis of key regulators in phytohormone pathways. However, there is no report on the role of E3 ubiquitin ligase in cold acclimation. The enrichment of several E3 ligases may indicate that the ubiquitination process was beneficial to the cold acclimation of *K. obovata* seedlings, but the substrates of these E3 ligases were not clear and required further investigation.

### 4.3 Transcriptional regulation in the cold acclimation of *K. obovata* seedlings

We found that four *koCBF*s were upregulated in response to cold waves (**Supplementary Figure 11**), with *koCBF3* and *koCBF4* being CARGs (**Supplementary Table 2**). CBFs are transcription factors that mediate plant cold tolerance by binding to CRT/DRE cis-elements containing the conserved ‘CCGAC’ sequence (Shi et al., 2018). About 43% of CARGs had at least one ‘CCGAC’ conserved binding motif of CBFs in their promoter region (**Supplementary Figure 12**). We found a previously validated CBF-regulating gene, *COR47* (Gilmour et al., 1992), containing four ‘CCGAC’ motifs (**Supplementary Table 2**), suggesting that our motif prediction approach yielded robust results. *koCBF4* was a hub transcription factor in the Module turquoise. These results indicated that the classic CBF-dependent pathway may function in the *K. obovata* seedlings’ cold acclimation process (**Figure 5**).

Many CBF-dependent genes known to play important roles in cold acclimation did not show significant changes in this study e.g. *ICE1*, *MPK4*, *MPK6*, or *OST1* (Shi et al., 2018). This is possible because this study adopted long-term cold waves, whereas the above genes reflect the immediate response to cold stress. These results suggest that cold acclimation and transient cold response might be underlined by different molecular mechanisms.

Besides CBFs, we found several other hub-transcription factors, such as *CZF1*s, *ZAT*s in regulating the co-expression during cold acclimation (**Figure 4D**, **5**). The findings of these transcription factors are consistent with previous studies that cold acclimation can be achieved by CBF-independent pathways (Park et al., 2018). The upregulated expression of *CZF1*s and *ZAT*s under cold acclimation is consistent with reports in *A. thaliana* and other woody plants (Park et al., 2018; Liang et al., 2020). The study of *A. thaliana* showed that the CBF-independent pathway may co-regulate the downstream cold response genes (Park et al., 2018). As the CBF motif is only observed on about half of CARGs (**Figure S6**, **Supplementary Table 3**), CBF-independent transcription factors are anticipated to play an equally important role in regulating the cold acclimation of *K. obovata* seedlings (**Figure 5**).

## 5 Conclusion

In this study, we found the mangrove species *K. obovata* seedlings adopted a cold acclimation mechanism in response to repeat cold waves. The cold acclimation mechanism involves reprogramming genes encoded signaling factors, transcription factors, and downstream enzymes involved in cell wall remodeling and regulation of ubiquitination. Such a cold acclimation process may help *K. obovata* seedlings survive the winter in north subtropical regions. In the future, more in-depth gene function verification is required to explore how these transcription factors and the downstream genes they regulate make the *K. obovata* form cold acclimation, whether they are CBF-dependent or -independent. More importantly, field planting experiments should be carried out to verify whether the seedlings acclimated to cold will demonstrate improved abilities in cold tolerance. Identifying mangroves’ cold acclimation mechanism will provide critical insights into the conservation of mangrove forests.

## Data availability statement

The datasets presented in this study can be found in online repositories. The names of the repository/repositories and accession number(s) can be found below: https://www.ncbi.nlm.nih.gov/, PRJNA678025.

## Author contributions

YS conceived and designed the project. SH, XW, YFZ, LW, PL, and ZD participated in sampling, physiological indices detection, sequencing, and/or data analyses. Y-YZ involved in experimental design and data analysis. YS and SH wrote the manuscript and all authors critically revised the manuscript. All authors contributed to the article and approved the submitted version.

## Footnotes

1 WorldClim. https://worldclim.org/data/index.html [Accessed December 29, 2021].

2 OmicShare. https://www.omicshare.com/tools [Accessed March 03, 2021].
