# Supplementary material for: Physiological and transcriptomic responses to cold waves of the most cold-tolerant mangrove, Kandelia obovata
Source: Front Plant Sci. 2023 Feb 10;14:1069055. doi: 10.3389/fpls.2023.1069055 (PMC9950753; doi:10.3389/fpls.2023.1069055)
Supplement: Supplementary file 1 [file DataSheet_1.pdf]

Supplementary Material

1 Supplementary Information I

```
#!/usr/bin/perl -w

use strict;

use warnings;

use Getopt::Long;

use Data::Dumper;

use Config::General;

use Cwd qw(abs_path getcwd);

use FindBin qw($Bin $Script);

use File::Basename qw(basename dirname);

use Bio::SeqIO;

use Bio::Seq;

my $version = "1.3";


##prepare                                                                parameters
##########################################################################

## GetOptions


my %opts;

GetOptions(\%opts,  "gff=s","fa=s", "out=s", "n=s","h");

if(!defined($opts{out}) | !defined($opts{gff}) || !defined($opts{fa}) | defined($opts{h}))

{

print <<"Usage End.";

Description:
```

```
$version:lefse analysis

Usage

Forced parameter:

-gff        gff file                <infile>    must be given

-out        outdir                  <outfile>      must be given

-n      num     <int>

-fa     genome fasta file   <infile>    must be given

Other parameter:

-h          Help document

Usage End.

exit;

}

$opts{n} ||= 2000;

my $n = $opts{n};

my $in  = Bio::SeqIO->new(-file => "$opts{fa}" , -format => 'Fasta');

my %fasta;

while ( my $seq = $in->next_seq() ) {

my($id,$sequence)=($seq->id,$seq->seq);

$fasta{$id}=$sequence;

}

open(IN,"$opts{gff}") ||die "open file $opts{gff} faild.\n";

open(OUT,">$opts{out}") ||die "open file $opts{out} faild.\n";

while(<IN>){

next if(/^#/);

my @line = split ("\t",$_);
```

```
if($line[2] eq "gene"){
$line[8] =~ /ID=([^;]*);/;
my $name = $1;
if($line[6] eq "+"){
my $gene = substr( $fasta{$line[0]},$line[3]-$n-1, $n);
print OUT ">$name\n$gene\n";


}elsif($line[6] eq "-"){
my $gene = substr( $fasta{$line[0]},$line[4], $n);
$gene = &reverse_complement_IUPAC($gene);
print OUT ">$name\n$gene\n";
}
}
}
close(OUT);
close(IN);
sub reverse_complement_IUPAC {
	my $dna = shift;
	# reverse the DNA sequence
	my $revcomp = reverse($dna);
	# complement the reversed DNA sequence
	$revcomp =~
tr/ABCDGHMNRSTUVWXYabcdghmnrstuvwxy/TVGHCDKNYSAABWXRtvghcdknysaabwxr/;
	return $revcomp;
}
sub reverse_complement {
```

```
    my $dna = shift;
    # reverse the DNA sequence
    my $revcomp = reverse($dna);
    # complement the reversed DNA sequence
    $revcomp =~ tr/ACGTacgt/TGCAtgca/;
    return $revcomp;
}
```

2 Supplementary Figures

(**A**)

(**B**)

(**C**)

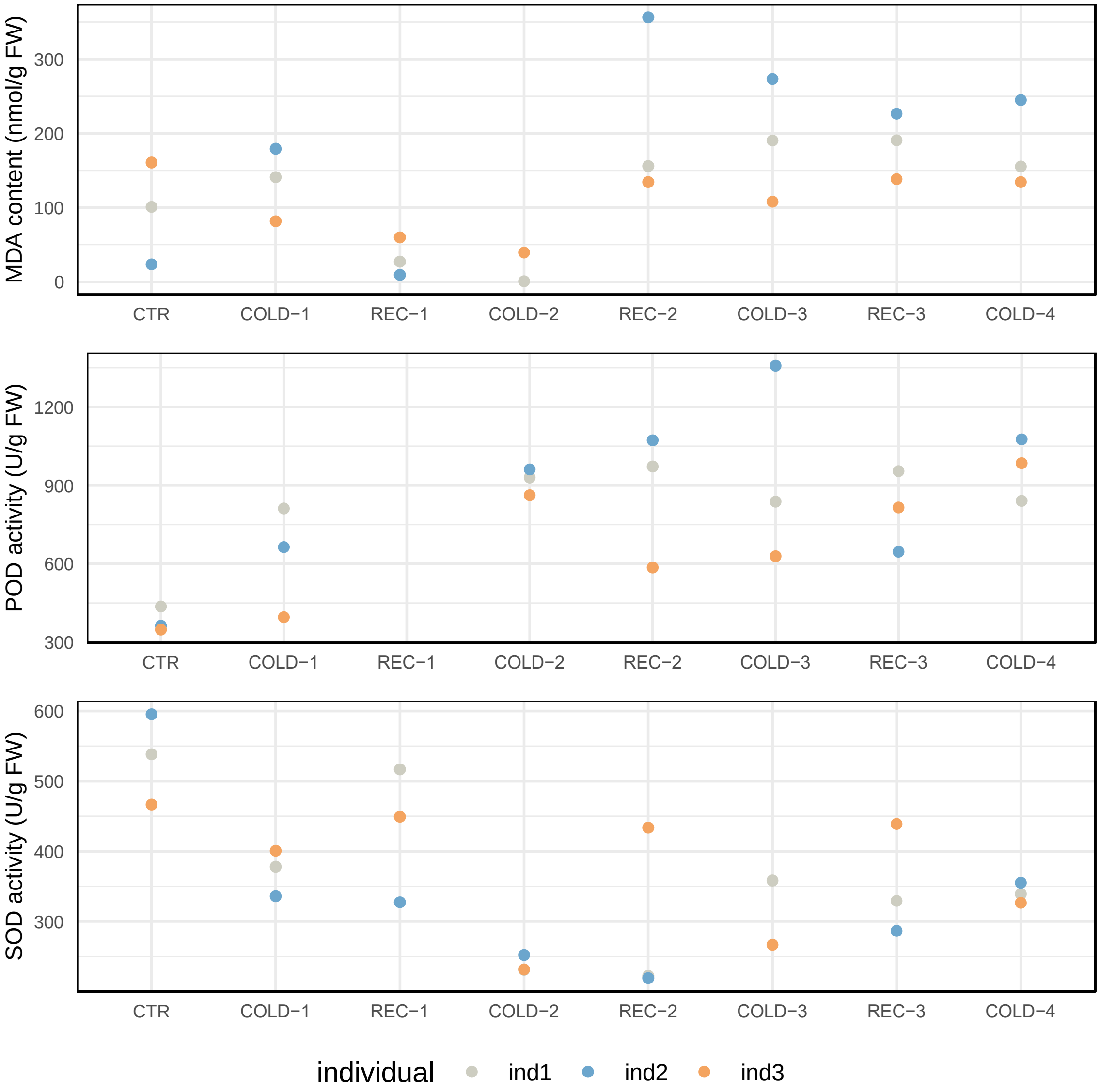

Supplementary Figure 1. Changes of MDA content (**A**), POD activity (**B**), and SOD activity (**C**)

(**A**)

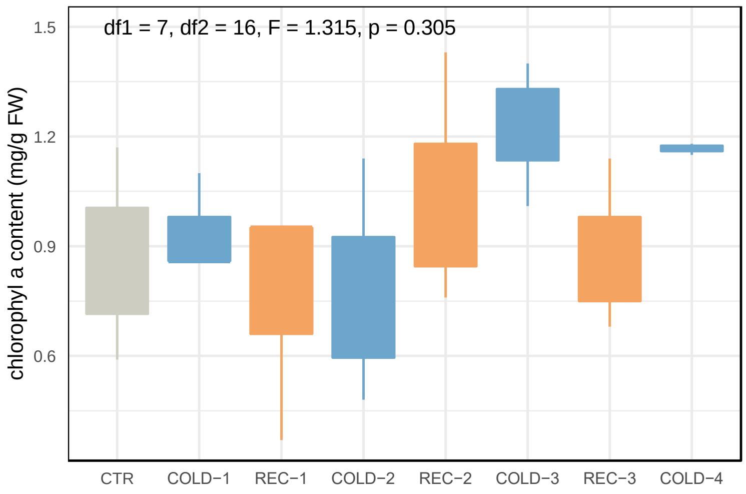

(**B**)

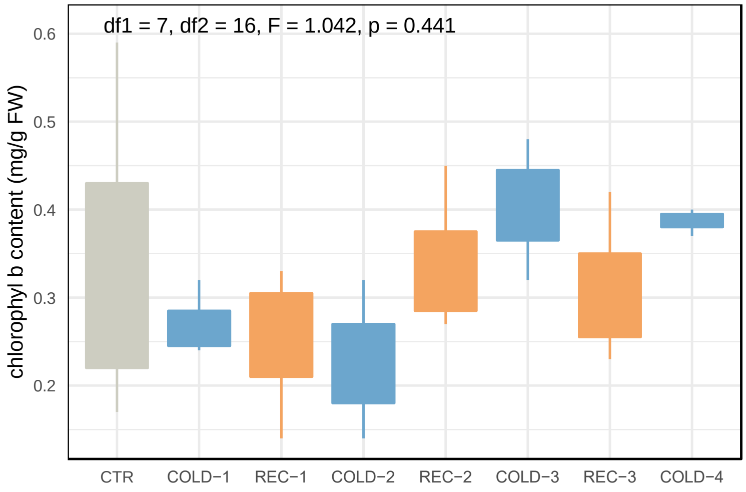

Supplementary Figure 2. Changes of Chlorophyll a (**A**) and Chlorophyll b (**B**) contents

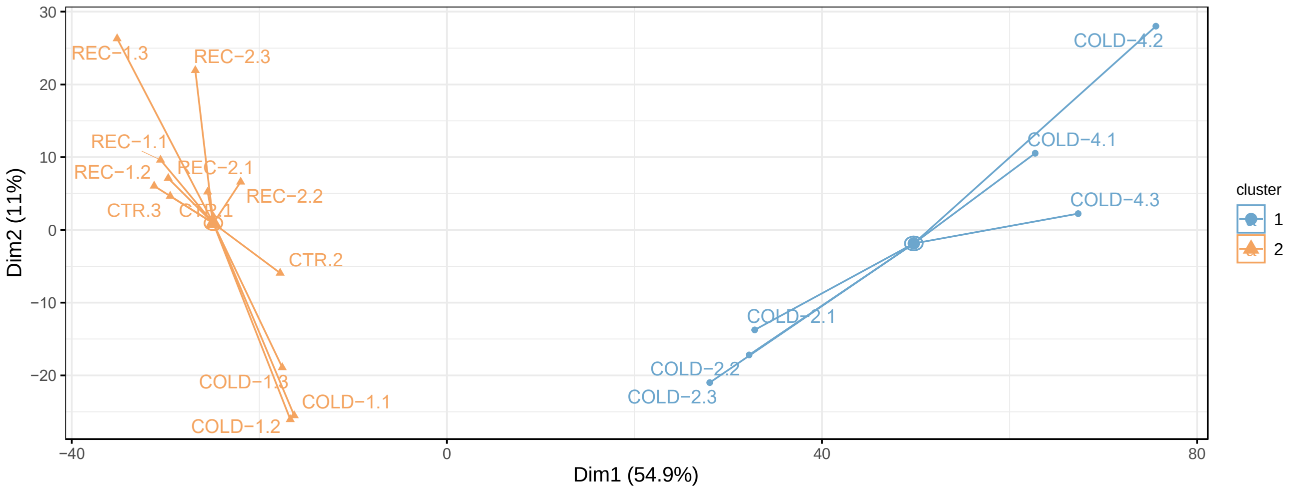

Supplementary Figure 3. *k*-means results of transcriptome data

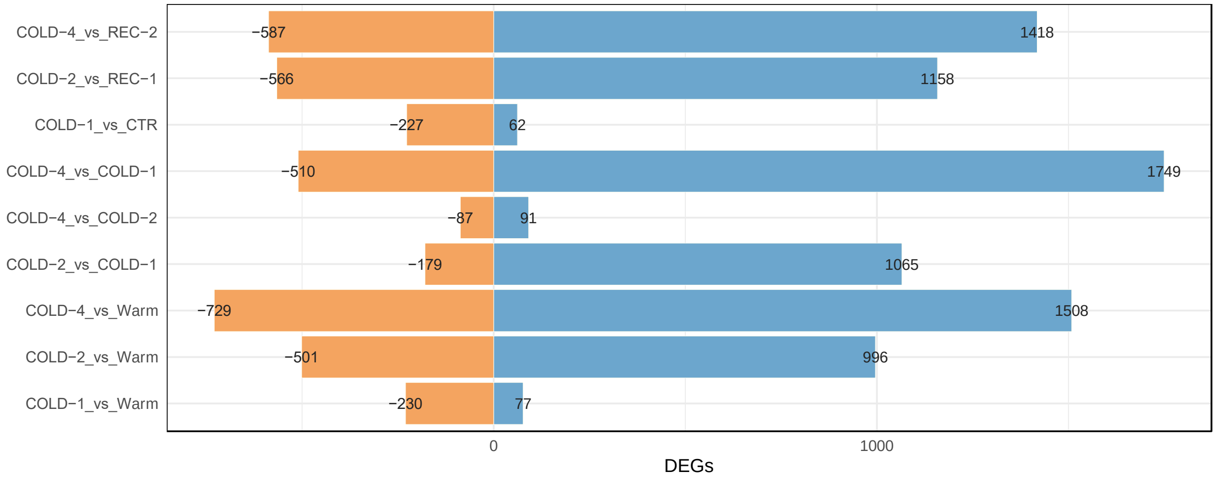

Supplementary Figure 4. Up and down regulated DEGs in pairwise comparisons

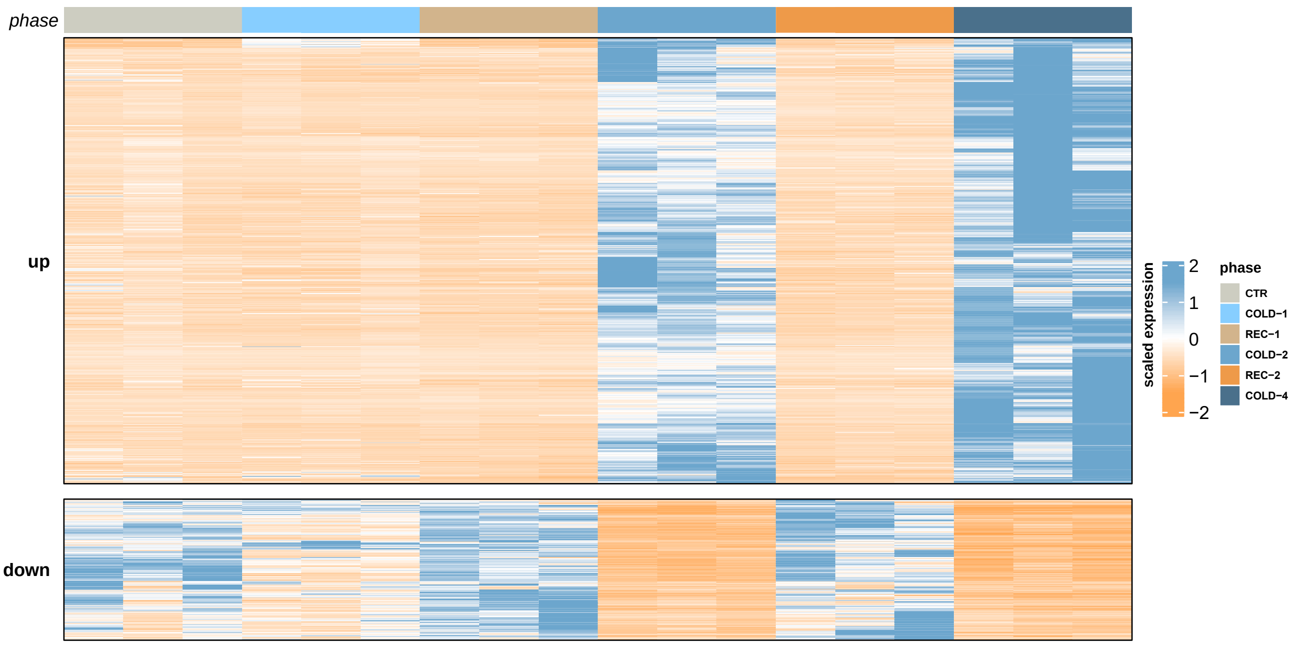

Supplementary Figure 5. The expression of all CARGs

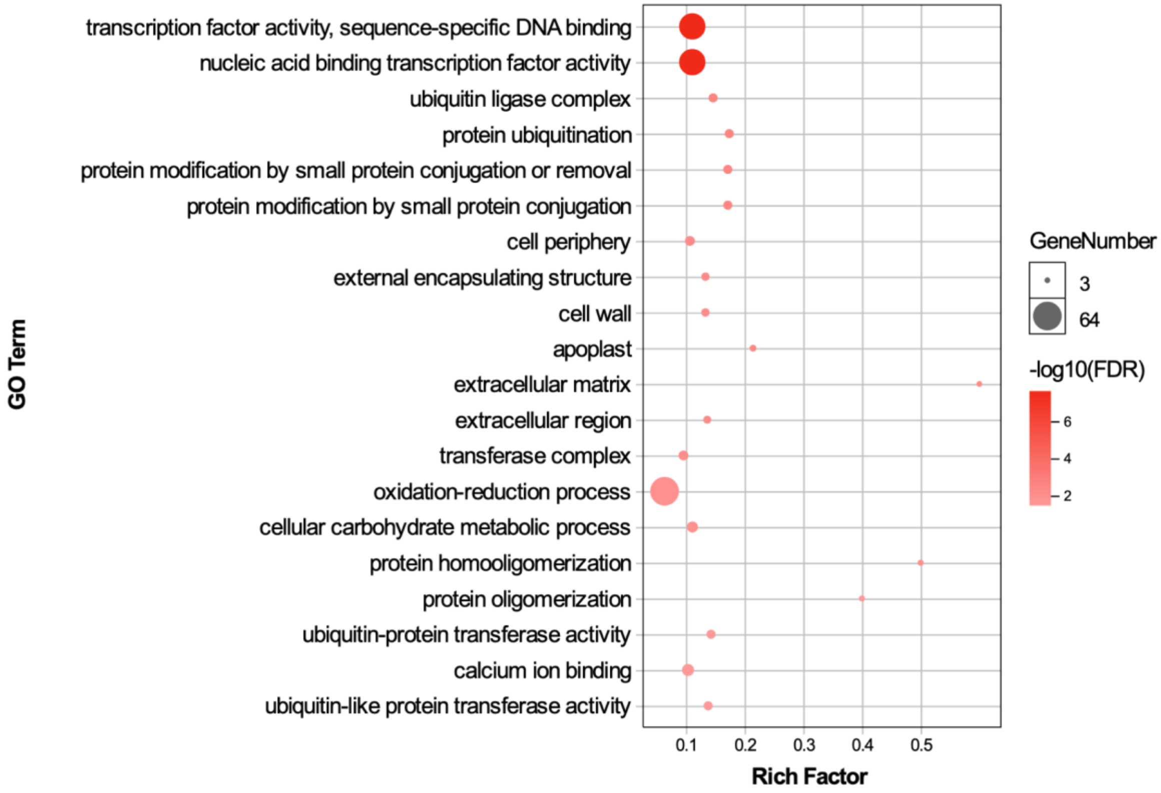

Supplementary Figure 6. The Gene Ontology enrichment results of all CARGs

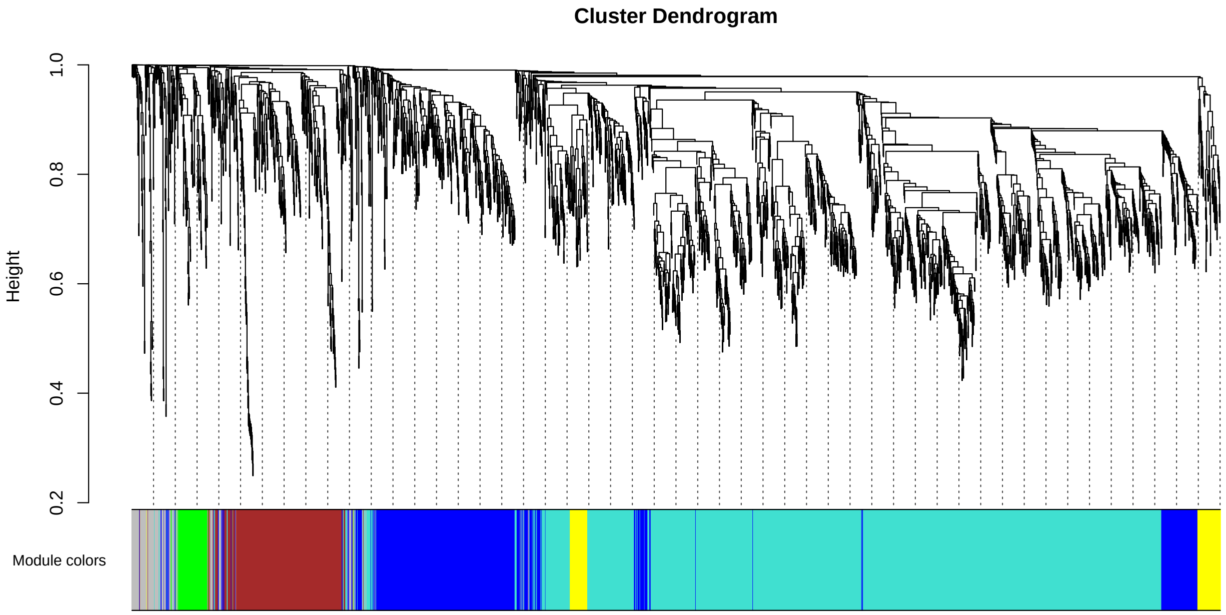

Supplementary Figure 7. Hierarchical cluster dendrogram of gene co-expression modules of DEGs using the weighted gene correlation network analysis (WGCNA) method

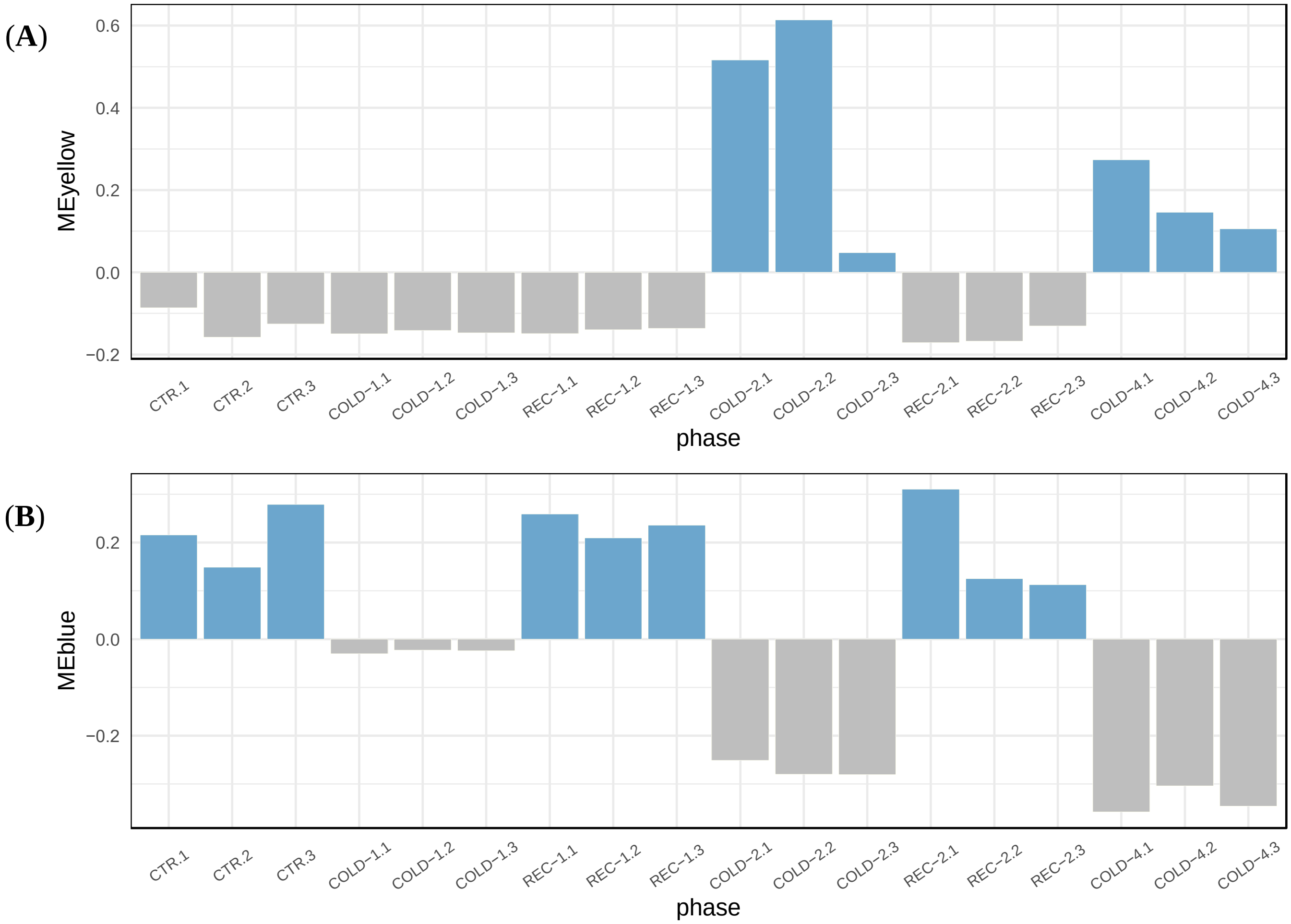

Supplementary Figure 8. Expression profiles of eigengenes in Modules yellow and blue

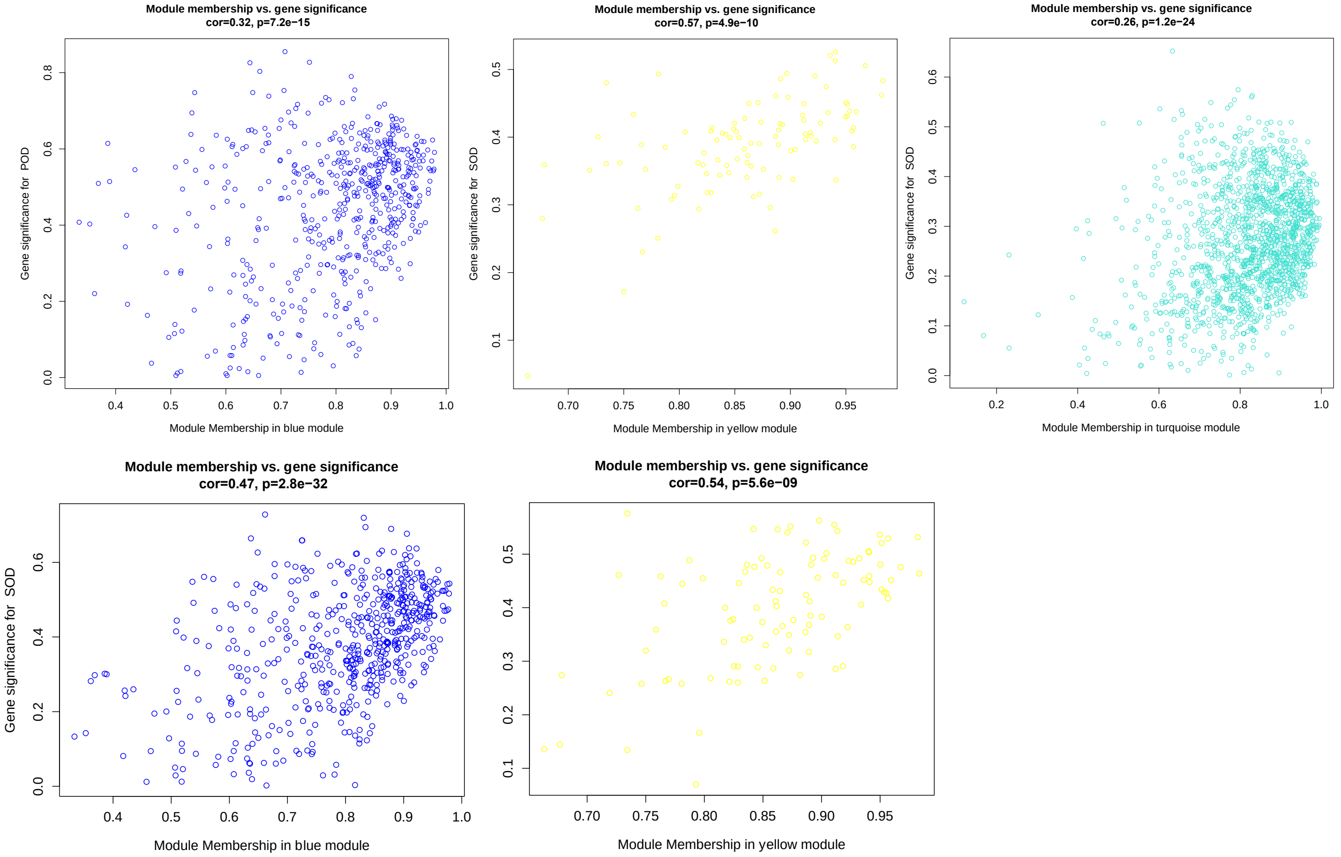

Supplementary Figure 9. The gene significance for physiological traits (y-axis) vs. the module membership (x-axis) plotted separately in modules turquoise, blue and yellow

The module membership is the module eigengene-based network connectivity. Gene significance for SOD/POD means a SOD/POD activity-based gene significance obtained by correlating the modules to SOD/POD activity values with the eigengene network methodology. We observed that the within-module hub genes in module turquoise, yellow and blue showed a positive correlation between gene significance and module membership.

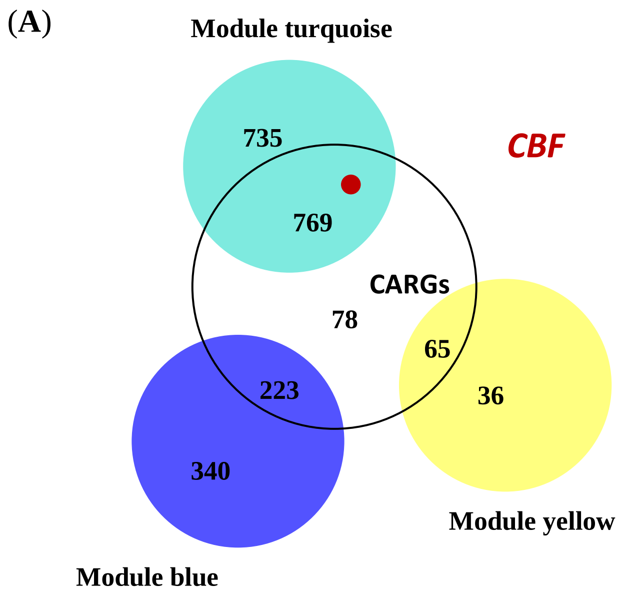

Supplementary Figure 10. CARGs vs. genes in Modules turquoise, blue and yellow

(**A**) All CARGs vs. all genes in Modules turquoise, blue and yellow. (**B**) All CARGs vs. all hub genes in Modules turquoise, blue and yellow.

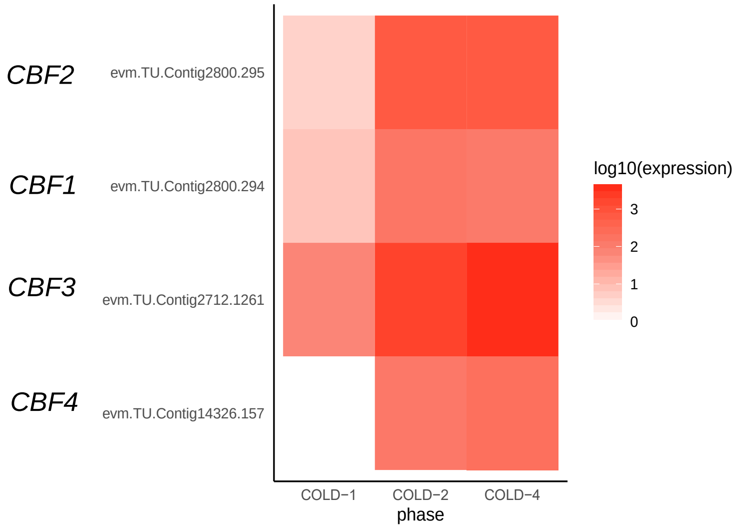

Supplementary Figure 11. The expression of all *koCBF*s

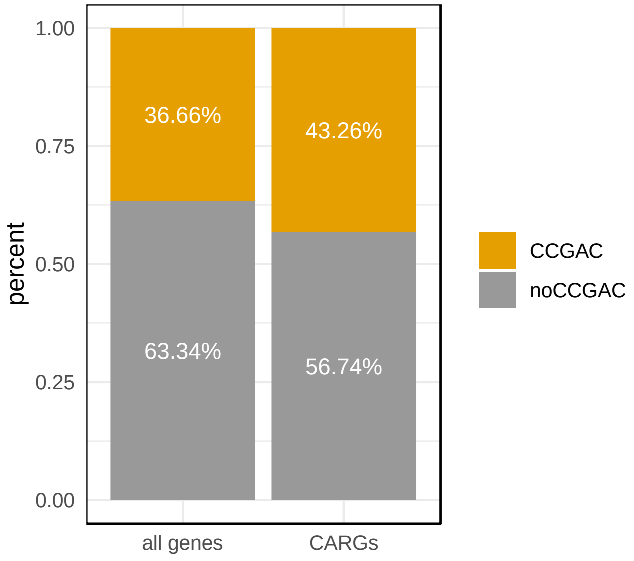

Supplementary Figure 12. The proportions of genes contained 'CCGAC' sequences in the promotor region among CARGs versus among all *K. obovata* genes

CBF can regulated downstream COR genes by binding to CRT/ DRE cis-elements containing the conserved CCGAC sequence in their promoters. To investigate the possible *koCBF* target genes, an in-house Perl scripts was used to extract the upstream 2000 bp as putative promoter regions of all *K. obovata* genes to find the existence of the CRT/DRE motif 'CCGAC'. 37% genes have at least one CCGAC sequence among all *K. obovata* genes, but the percentage was up to 43% among all CARGs.

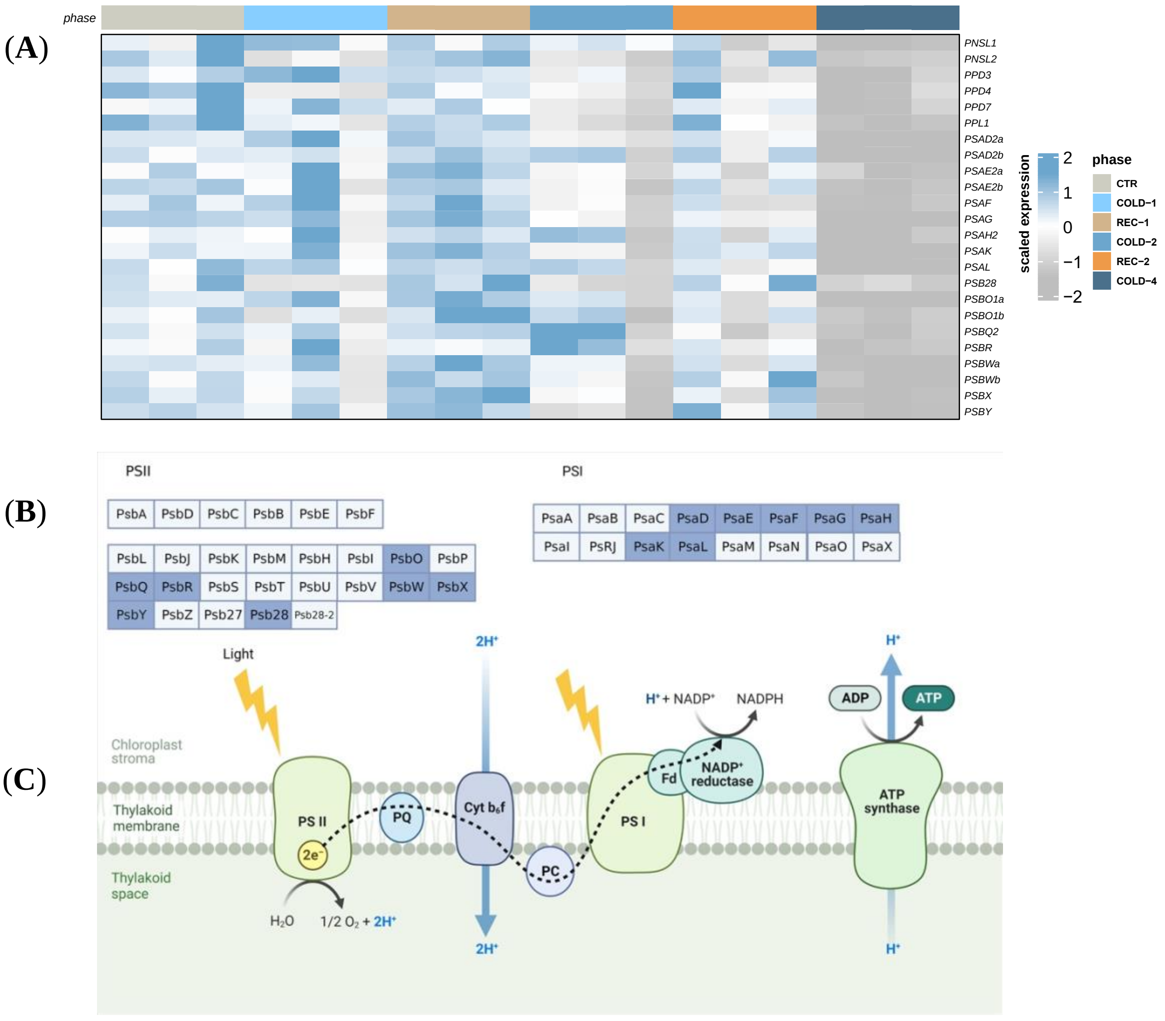

Supplementary Figure 13. The differential expression of genes related to the photosystem

(**A**) Expression changes of DEGs (COLD-4_vs_Warm) related to photosystem. (**B**) Subunits of PSI and PSII. Blue and white represent downregulated gene and nonregulated gene, respectively. (**C**) Schematic diagram of plant photosystem, modified based on the KEGG pathway (map00195).
